# Use of low-dose computed tomography to assess pulmonary tuberculosis among healthcare workers in a tuberculosis hospital

**DOI:** 10.1186/s40249-017-0274-6

**Published:** 2017-03-24

**Authors:** Wei He, Bu-Dong Chen, Yan Lv, Zhen Zhou, Jin-Ping Xu, Ping-Xin Lv, Xin-Hua Zhou, Feng-Gang Ning, Cheng-Hai Li, Dong-Po Wang, Jie Zheng

**Affiliations:** 1 Beijing Chest Hospital, Capital Medical University, No. 97 Beimachang, Tongzhou District, Beijing, 101149 China; 2 The school hospital, Beijing Science and Technology University, Beijing, 100083 China; 3 Mallinckrodt Institute of Radiology, Washington University School of Medicine, 4525 Scott Ave, Room 3114, Saint Louis, MO 63110 USA

**Keywords:** Tuberculosis, Pulmonary, Active tuberculosis, Healthcare workers, Low-dose computed tomography, Computed tomography

## Abstract

**Background:**

According to the World Health Organization, China is one of 22 countries with serious tuberculosis (TB) infections and one of the 27 countries with serious multidrug-resistant TB strains. Despite the decline of tuberculosis in the overall population, healthcare workers (HCWs) are still at a high risk of infection. Compared with high-income countries, the TB prevalence among HCWs is higher in low- and middle-income countries. Low-dose computed tomography (LDCT) is becoming more popular due to its superior sensitivity and lower radiation dose. However, there have been no reports about active pulmonary tuberculosis (PTB) among HCWs as assessed with LDCT. The purposes of this study were to examine PTB statuses in HCWs in hospitals specializing in TB treatment and explore the significance of the application of LDCT to these workers.

**Methods:**

This study retrospectively analysed the physical examination data of healthcare workers in the Beijing Chest Hospital from September 2012 to December 2015. Low-dose lung CT examinations were performed in all cases. The comparisons between active and inactive PTB according to the CT findings were made using the Pearson chi-square test or the Fisher’s exact test. Comparisons between the incidences of active PTB in high-risk areas and non-high-risk areas were performed using the Pearson chi-square test. Analyses of active PTB were performed according to different ages, numbers of years on the job, and the risks of the working areas. Active PTB as diagnosed by the LDCT examinations alone was compared with the final comprehensive diagnoses, and the sensitivity and positive predictive value were calculated.

**Results:**

A total of 1 012 participants were included in this study. During the 4-year period of medical examinations, active PTB was found in 19 cases, and inactive PTB was found in 109 cases. The prevalence of active PTB in the participants was 1.24%, 0.67%, 0.81%, and 0.53% for years 2012 to 2015. The corresponding incidences of active PTB among the tuberculosis hospital participants were 0.86%, 0.41%, 0.54%, and 0.26%. Most HCWs with active TB (78.9%, 15/19) worked in the high-risk areas of the hospital. There was a significant difference in the incidences of active PTB between the HCWs who worked in the high-risk and non-high-risk areas (odds ratio [*OR*], 14.415; 95% *confidence interval* (*CI*): 4.733 – 43.896). Comparisons of the CT signs between the active and inactive groups via chi-square tests revealed that the tree-in-bud, cavity, fibrous shadow, and calcification signs exhibited significant differences (*P* = 0.000, 0.021, 0.001, and 0.024, respectively). Tree-in-bud and cavity opacities suggest active pulmonary tuberculosis, whereas fibrous shadow and calcification opacities are the main features of inactive pulmonary tuberculosis. Comparison with the final comprehensive diagnoses revealed that the sensitivity and positive predictive value of the diagnoses of active PTB based on LDCT alone were 100% and 86.4%, respectively.

**Conclusions:**

Healthcare workers in tuberculosis hospitals are a high-risk group for active PTB. Yearly LDCT examinations of such high-risk groups are feasible and necessary.

**Electronic supplementary material:**

The online version of this article (doi:10.1186/s40249-017-0274-6) contains supplementary material, which is available to authorized users.

## Multilingual abstracts

Please see Additional file 1 for translations of the abstract into the five official working languages of the United Nations.

## Background

Active tuberculosis (TB) is an infectious disease caused by the bacteria *Mycobacterium tuberculosis* (*M. tuberculosis*) and can spread from person to person through the air. *M. tuberculosis* complex are transmitted by droplet infection in addition to rarely being spread via smear infections on the skin and mucous membranes, via contaminated dust particles or cuts and stab wounds involving contaminated cannulae or scalpels [1].

TB is a major global health problem. There were an estimated 9.6 million incident cases of TB in 2014 of which 5.4 million were adult men, 3.2 million were adult women, and 1.0 million were children [2]. Moreover, there were an estimated 1.5 million deaths from TB [2]. The number of TB cases relative to population size (i.e., the incidence rate) varies widely among countries. The TB burden is expressed in terms of the estimated annual incidence, mortality, and disease prevalence. These three outcome measures are reported per 100 000 people. Estimates from the World Health Organization (WHO) are derived from population-based national surveys of the prevalence of TB, time series of case notifications, and mortality data from vital registration systems with the standard coding of the causes of death. Most high-TB burden countries that collectively account for 80% of TB cases have rates of approximately 150 – 300 cases per 100 000 population per year [3]. According to the WHO, China is one of the 22 countries with serious TB infection and one of the 27 countries with serious multidrug-resistant TB strains [3]. In 2014, the number of TB infections in China was approximately 930 000, which accounted for nearly 10% of all global infections [2]. A total of 147 941 TB cases were reported from 2009 to 2012 in Zhejiang Province alone [4].

Globally, TB prevalence in 2015 was 42% lower than that in 1990 [2]. However, despite the decline of TB in the overall population, healthcare workers (HCWs) remain at a high risk of infection [5]. A subsequent meta-analysis estimated that the average annual risk of developing TB disease was three times higher (95% *CI*: 2.43 – 3.51) for HCWs (across all settings) compared to the general population [6]. The median annual incidence of TB among HCWs was 67, 91, and 1 180 cases/100 000 persons in studies from countries with low, intermediate, and high TB incidence, respectively. The corresponding median TB incidences for the general populations were 33, 82, and 311 cases/100 000 persons [5]. Compared with high-income countries, the TB prevalence among HCWs from low- and middle-income countries are higher [7, 8]. A systematic review of the TB incidence in low- and middle-income countries estimated that the annual risk of TB infection among HCWs ranges from 3.9% to 14.3% (with between 2.6% and 11.3% of cases attributable to occupational exposure) [7]. The early detection and treatment of TB in HCWs has important clinical significance.

Few studies have evaluated the risk of active pulmonary TB (PTB) among HCWs in China. It has been reported that tuberculin skin test (TST) reactions ≥ 5 mm occur in 69% of the HCWs in Inner Mongolia, China [9]. A prospective cohort study that enrolled HCWs in a tertiary general hospital in Beijing, China revealed that 29/101 HCWs (28.7%) received positive diagnoses based on T-SPOT. TB and 53/101 (55.2%) were positive according to TST (using a ≥ 10 mm cut-off) [10]. At present, active TB detection in high-risk groups primarily relies on X-ray. Low-dose CT (LDCT) is becoming more popular due to its superior sensitivity and lower radiation dose. However, there has been no reporting regarding active pulmonary tuberculosis among HCWs as detected by LDCT. The purpose of this study is to examine the active PTB statuses of HCWs in hospitals specializing in TB treatment. In this project, the physical examination results from HCWs working at the Beijing Chest Hospital over a period of 4 years (2012 – 2015) were retrospectively analysed. These analyses included all LDCT scanning data.

## Methods

### Participants

We retrospectively analysed the health examination data of healthcare workers from the Beijing Chest Hospital affiliated with Capital Medical University from January 2012 to November 2015. The inclusion criterion were the following: 1) at least one LDCT examination; 2) age > 18 years old; 3) If a HCW underwent 2 to 4 LDCT examinations within the four years, that HCW was considered 1 participant; and 4) if a HCW was diagnosed as a TB patient in a particular year, he/she was rescanned in the next year, but he/she was counted as one new case only in the year of the initial diagnosis year. The exclusion criteria were as follows: 1) CT examination with the conventional dosage and 2) During pregnancy. This study was approved by the Medical Ethics Committee of the Beijing Chest Hospital affiliated with Capital Medical University. All participants provided written informed consent before participating in the study.

### Low-dose CT image acquisition

All LDCT scans were performed using an Optima CT 680 Quantum or Lightspeed VCT (General Electric Company, GE, America) scanners. The scanning scheme involved employed two types. Four hundred participants were scanned with a fixed tube voltage (120 kV) and current (50 mA), and 612 participants were scanned with automatic dose adjustment. For the latter type, the dose range was 10 – 80 mA, and the average tube voltage was 120 kV. The scans were performed with spiral data acquisition and the following additional acquisition parameters: pitch, 1.375; and noise factor, 25. For the LDCTs, all the images were reconstructed into axial images with 5 mm slice thickness at 5 mm intervals with a lung and mediastinal window algorithm and a 1.25 mm thin-section lung window using a lung reconstruction algorithm.

### Image analysis and classification of TB

All CT scans and medical records were retrospectively reviewed by two chest radiologists with 16 and 20 years of experience. The two radiologists independently and blindly analysed the CT scans for the presence or absence and extent of the TB features. The resolution of differences in the observed findings was based on a consensus between the two radiologists. The airway abnormalities, lung abnormalities, pleura abnormalities, and lymph node enlargements were recorded.

According to the literature [11–13], the signs of PTB activity on CT include the following: tree-in-bud and centrilobular nodules, lobular or segmental or lobar/subsegmental consolidations, ground glass opacities, thick wall cavities, bronchial wall thickenings and masses (>3 cm). The signs of inactive PTB were fibrous and calcified lesions. Combined with the guidelines of the Health of the People’s Republic of China Industry Standards for Tuberculosis [14], the PTB diagnostic criteria for the physical examinations are provided in Table 1.

**Table 1:** Criteria for TB diagnosis in the physical examination

Classification	Definition	Diagnosis
Active PTB	(1) smear-positive and culture-positive tuberculosis in sputum and bronchial lavage fluid; pathological diagnosis for tuberculosis in the lung lesions (2) although 3 sputum smear-negative tests, chest radiographic examination showing active tuberculosis in the lesions (3) tuberculosis suspicious symptoms such as cough, expectoration, hemoptysis; (4) strong positive tuberculin test; (5) anti-tuberculosis antibody examination positive; (6) extrapulmonary histopathological examination confirming tuberculosis; (7) anti-inflammatory therapy invalid and antituberculosis diagnostic treatment or follow-up show valid.	(1) or (2) + (3)–(7) in any of the terms
Inactive PTB	(1) residual lesions are stable for more than six months or gradual fibrosis and calcification occurs in persons who were previously diagnosed with active tuberculosis and cured by effective anti-tuberculosis treatment; (2) lack of a history of active tuberculosis diagnosis, no symptoms such as cough, sputum, or hemoptysis and sputum tuberculosis bacterium negative but chest CT showed lesions that conforms to PTB that in common locations of pulmonary tuberculosis, and the lesions did not change for more than 6 months without anti-tuberculosis treatment.	(1) or (2)

### Classification of the work areas

High-risk areas were defined as the working areas in the hospital in which diagnosed or undiagnosed patients with TB were likely to be cared for. These areas included TB wards, multidrug-resistant TB wards, outpatient departments, TB outpatient clinics, general wards, and radiography clinics. Intermediate-risk areas were defined as working areas in the hospital in which there was a probability of having contact with patients with TB and included laboratories. The low-risk areas were defined as the working areas in the hospital in which there was little or no probability of having contact with patients and included administrative offices, finance departments, and libraries.

### Statistical analysis

The LDCT examination results were analysed using the Statistical Package for Social Sciences (SPSS) version 19.0. The comparisons between active and inactive PTB based on the CT findings were performed using the Pearson chi-square test or the Fisher’s exact test. Comparisons between the incidences of active PTB in the high-risk areas and the non-high-risk areas were performed using the Pearson chi-square test. The analyses of active PTB were performed according to different ages (20 – 25, 26 – 30, 31 – 35, 36 – 40, 41 – 45, 46 – 50, 51 – 55, 56 – 60, 55 – 60, and > 61 years old), different numbers of years on the job (0 – 5, 6 – 10, 11 – 15, 16 – 20, > 21), and different working risk areas (high risk, intermediate risk, and low risk areas). The active PTB diagnoses based on the LDCT examinations alone were compared with the final comprehensive diagnoses, and the sensitivity and positive predictive value were calculated. *P* values less than 0.05 were considered statistically significant.

## Results

### CT examination results

The numbers of HCWs in the hospital in 2012, 2013, 2014, and 2015 were 970, 970, 995, and 982, respectively. The numbers of participants in 2012, 2013, 2014, and 2015 were 809, 740, 743, and 752, respectively. A total of 1 012 participants were included in this study. There were 340 males and 672 females, and the average age was 40.57 ± 11.47 years old (18 – 82 years old). Of the 1012 participants, 243 participants were under 30 years old, 240 were between 31 and 40 years old, 334 were between 41 and 50, 169 were between 51 and 60, and 26 cases were older than 60 years.

### PTB statuses based on the LDCT examinations

From 2012 to 2015, 19 active PTB cases were found via LDCT examinations including 4 males and 15 females with a mean age of 39 years. Eighteen cases were diagnosed as secondary pulmonary tuberculosis including 2 sputum-positive bacteria and resistant tuberculosis cases and 1 case of mediastinal lymph node tuberculosis. The numbers of active PTB cases as assessed via annual CT examinations are provided in Table 2. The prevalence rates of active PTB in the participants were 1.24% (10/809), 0.67% (5/740), 0.81% (6/743), and 0.53% (4/752). The incidences of active PTB in the participants were 0.86% (7/809), 0.41% (3/740), 0.54% (4/743), and 0.26% (2/752). Based on comparisons with the final comprehensive diagnoses, the sensitivity and positive predictive value of the diagnoses of active PTB based on LDCT alone were 100% and 86.4%, respectively (Table 3). A total of 109 cases (10.9%) had inactive PTB over the four years.

**Table 2:** The numbers of active PTB participants diagnosed by LDCT examinations from 2012 ~ 2015

Year	Total subjects	Illness^c^	Incidence^e^	Post-primary PTB	Mediastinal LND TB^a^	Sputum positive	Sputum negative
2012	809	10^d^	7	10	0	1	9
2013	740	5	3	5	0	0	5
2014	743	6	4	5	1	1	5
2015	752	4	2	4	0	1^b^	3

^a^Mediastinal LND TB: mediastinal lymph node TB

^b^One patient was sputum-positive with active PTB and MDR-TB in 2014 and remained sputum-positive in 2015

^c^Current illness

^d^Ten cases exhibited pulmonary TB in 2012, including 7 newly discovered active tuberculosis cases and 3 previously diagnosed active tuberculosis cases who were receiving treatment cases

^e^Incidence: the number of people with active PTB that was newly diagnosed based on LDCT

**Table 3:** Comparison of the active PTB cases diagnosed based on LDCT examinations alone and based on the final comprehensive diagnosis

LDCT	Final Comprehensive diagnosis		Total
	Yes	No	
Yes	19	3	22
No	0	990	990
Total	19	993	1012

### Distributions of hospital participants with active pulmonary TB across the various departments

**Fig. 1:**
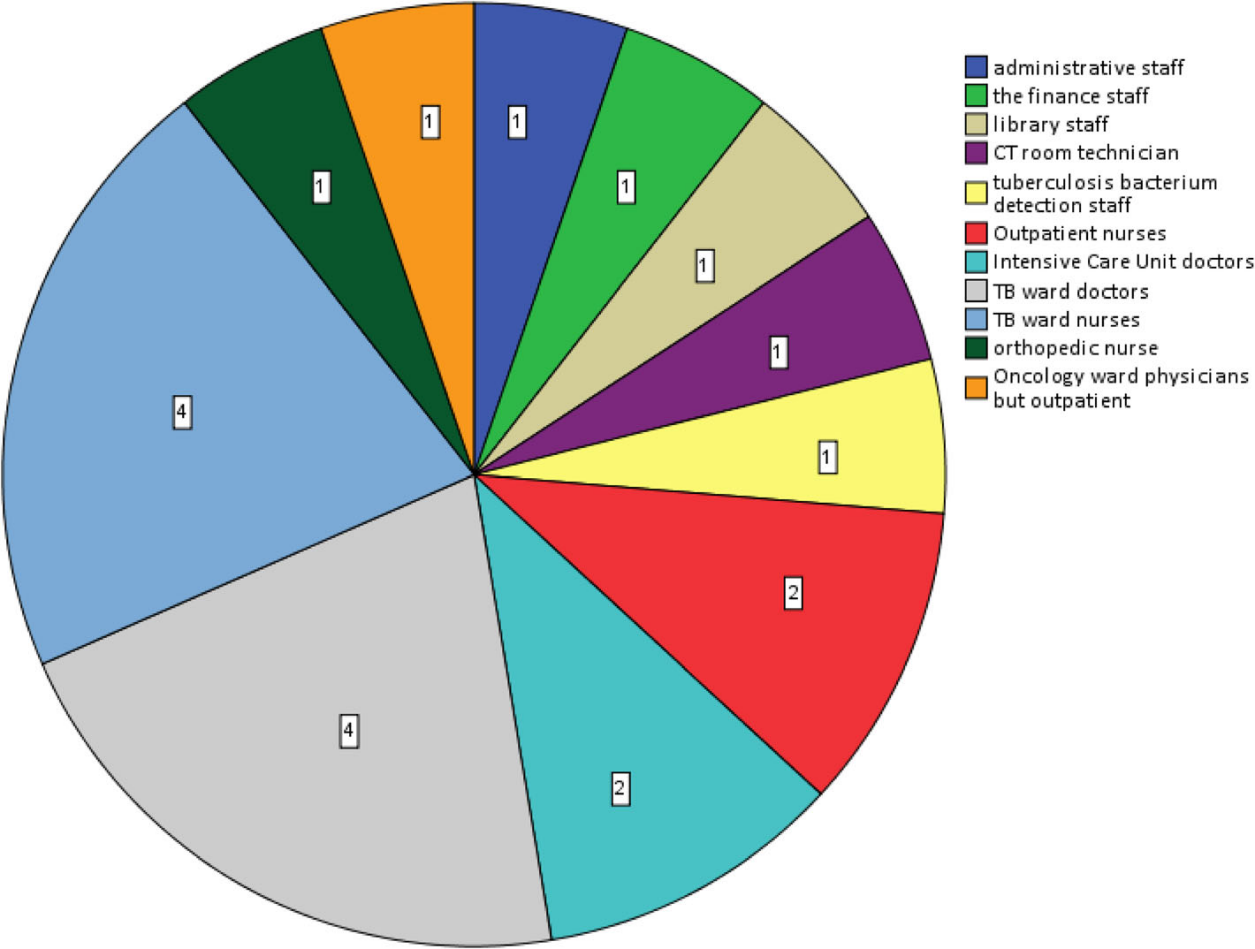
The population distributions of active pulmonary TB in the hospital staff across the various departments

Among the 19 active PTB cases, 3 (3/19, 15.8%) of the staff members did not work in clinical departments, and 2 (2/19, 10.5%) were paramedical staff who worked in laboratory conditions and as radiographers. However, 14 (14/19, 73.7%) were clinical staff (13 in the clinical departments that were in contact with TB patients and one in the oncology department, but this participant treated TB outpatients). The distributions active pulmonary TB in the hospital staff of the various departments are illustrated in Fig. 1, and the distributions of HCWs by age, years of employment, and working area are illustrated in Table 4. As indicated in Table 5, there was a significant difference in the incidence of active PTB between the HCWs who were working in the high-risk and non-high-risk areas (*χ* ^2^ = 33.901, *P* = 0.000; odds ratio [*OR*] = 14.415, 95% *CI*: 4.733 – 43.896).

**Table 4:** The numbers of active PTB cases from 2012 ~ 2015 separated according age, length of employment, and working area

	Number of active TB	Percentage of active TB(%)
Age (years)		
20–25	2	10.5
26–30	3	15.7
31–35	1	5.3
36–40	4	21.1
41–45	4	21.1
46–50	2	10.5
51–55	2	10.5
56–60	1	5.3
> 61	0	0
Length of employment (years)		
0–5	2	10.5
6–10	5	26.3
11–15	5	26.3
16–20	2	10.5
> 21	5	26.4
Work areas		
high-risk areas	15	78.9
intermediate-risk areas	1	5.3
low--risk areas	3	15.8

**Table 5:** Comparison of the incidences of active PTB cases in high-risk and non-high-risk areas

Areas	Active PTB		Total
	Yes	No	
High-risk	15	205	220
Non-high-risk	4	788	792
Total	19	993	1012

### CT signs of pulmonary TB according to the LDCT examinations

The main CT signs of active PTB discovered by LDCT from 2012 to 2015 are provided in Table 6. The comparison of the main CT signs discovered in the 19 active and 109 inactive PTB cases via LDCT is illustrated in Table 7. The comparisons between the two groups via chi-square tests revealed that the tree-in-bud (*P* = 0.000), cavity (*P* = 0.021), fibrous shadow (*P* = 0.001) and calcification (*P* = 0.024) signs exhibited significant differences. No significant differences were found for the other lesions.

**Table 6:** The features of active PTB on LDCT images from 2012 to 2015

	2012	2013	2014	2015
Tree-in-bud	3	1	1	2
Cavity	1	1	0	0
Patchy opacity	7	2	1	0
Calcification	1	0	0	0
Nodules	9	2	1	1
Ground glass opacity	3	1	0	0
Lymph node enlargement	0	0	1	0
Total	10	3	4	2

**Table 7:** Comparison of the features of active PTB and inactive PTB on LDCT images

CT sign	Active PTB	Inactive PTB
Tree-in-bud^a^	7	0
Cavity^a^	2	0
Patchy opacity	10	34
Calcification^a^	1	33
Nodules	13	75
Ground glass opacity	4	1
Lymph node enlargement	1	1
Fibrous shadow^a^	8	85
Total	19	109

^a^Significant difference between the active and inactive TB groups

**Fig. 2:**
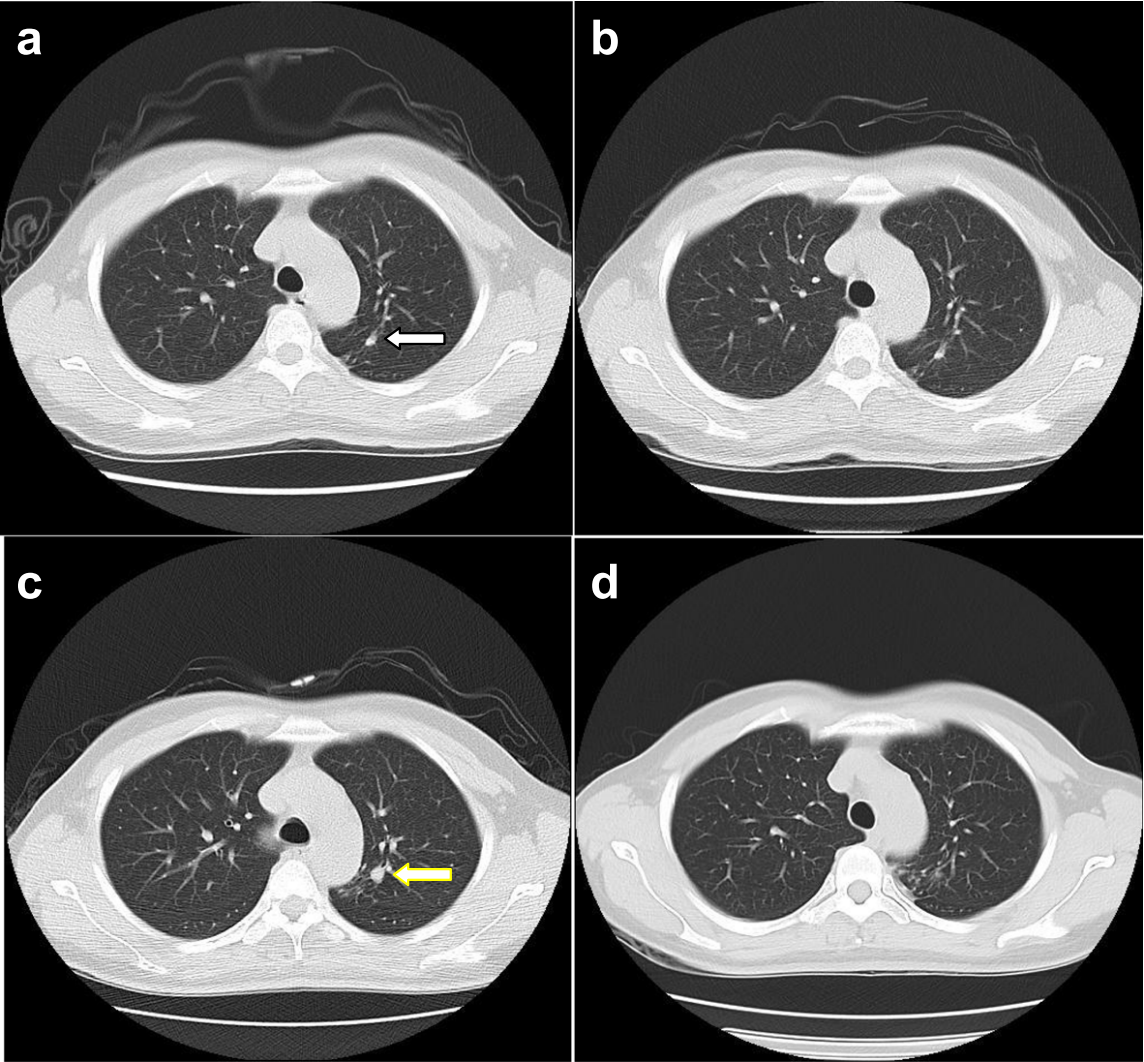
**a**-**d** LDCT images of an active PTB case (Case 1) from 2012 to 2015

A spiral CT in case 1 (Fig. 2a-d) illustrates an apicoposterior active, multidrug-resistant PTB in the left lung of a 42-year-old female patient. Panels A through D illustrate chest CT scan images collected from 2012 to 2015, respectively. Micronodules, ground glass opacity, and fibrous stripes shadow at the pulmonary window were observed in the left apicoposterior segment in 2012 (a, white arrow) and 2013 (b). Because there were no clinical symptoms, the TB was considered inactive. However, the micronodular shadows in the left upper apicoposterior segment grew with tree-in-bud opacities and micronodules shadow in 2014 (c, yellow arrow). The final diagnosis was active and multi-drug resistant TB. After a year of treatment, the micronodular shadows decreased in 2015 (d).

**Fig. 3:**
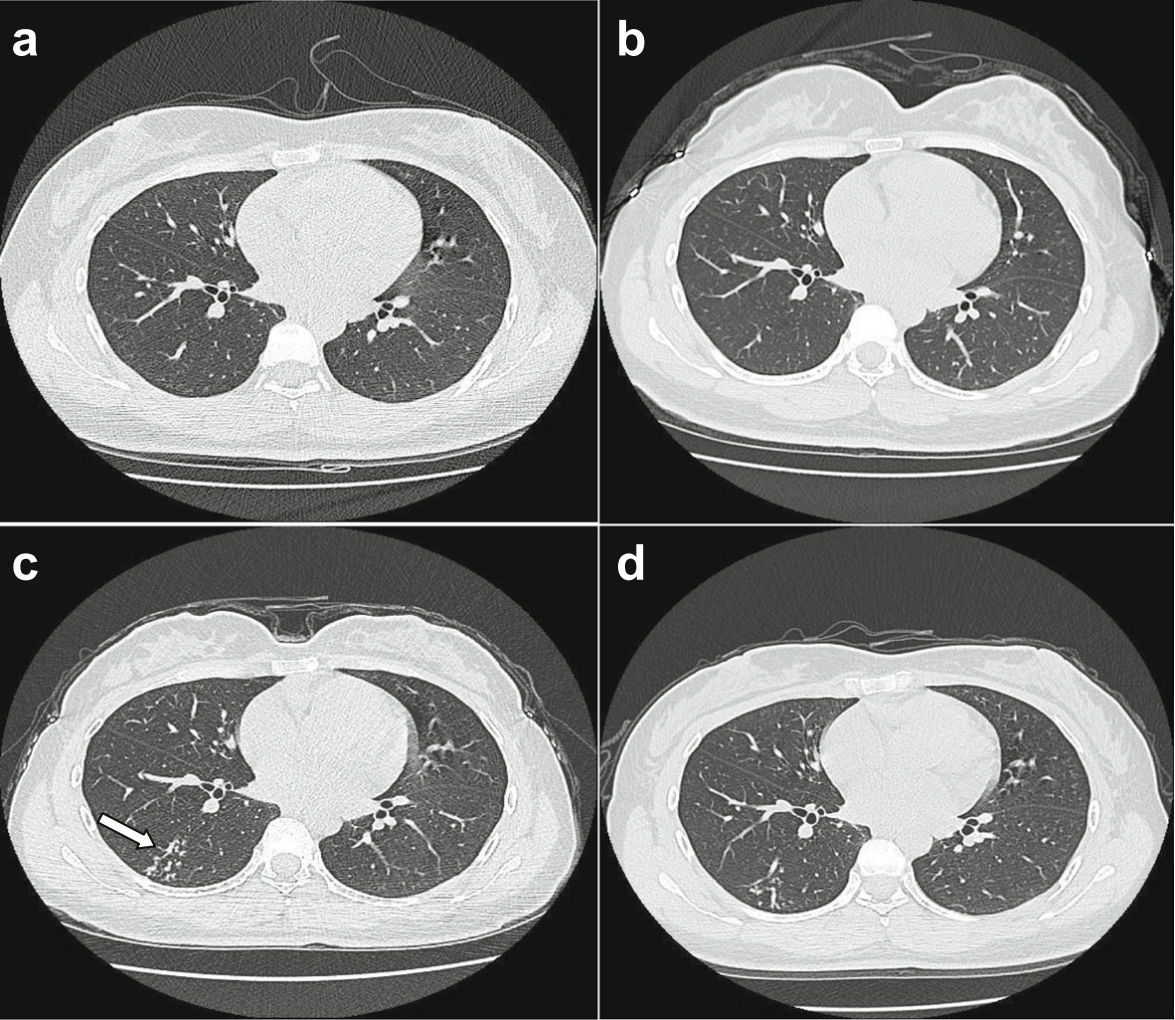
**a**-**d** LDCT images of an active PTB case (Case 2) from 2012 to 2015

A spiral CT from case 2 (Fig. 3a-d) illustrates a case of active PTB in the right lower lung in a 40-year-old female. The chest CT scan images in panels A through D were collected in 2012, 2013, and 2015, respectively. The lung was clear in 2012 (a) and 2013 (b). There was no physical examination in 2014. However, tree-in-bud nodules and centrilobular nodules in the pulmonary window (c, white arrow) were observed in the superior segment of the right lower lung in 2015. After three months of treatment, the tree-in-bud nodules and the lobular center nodules decreased (d).

**Fig. 4:**
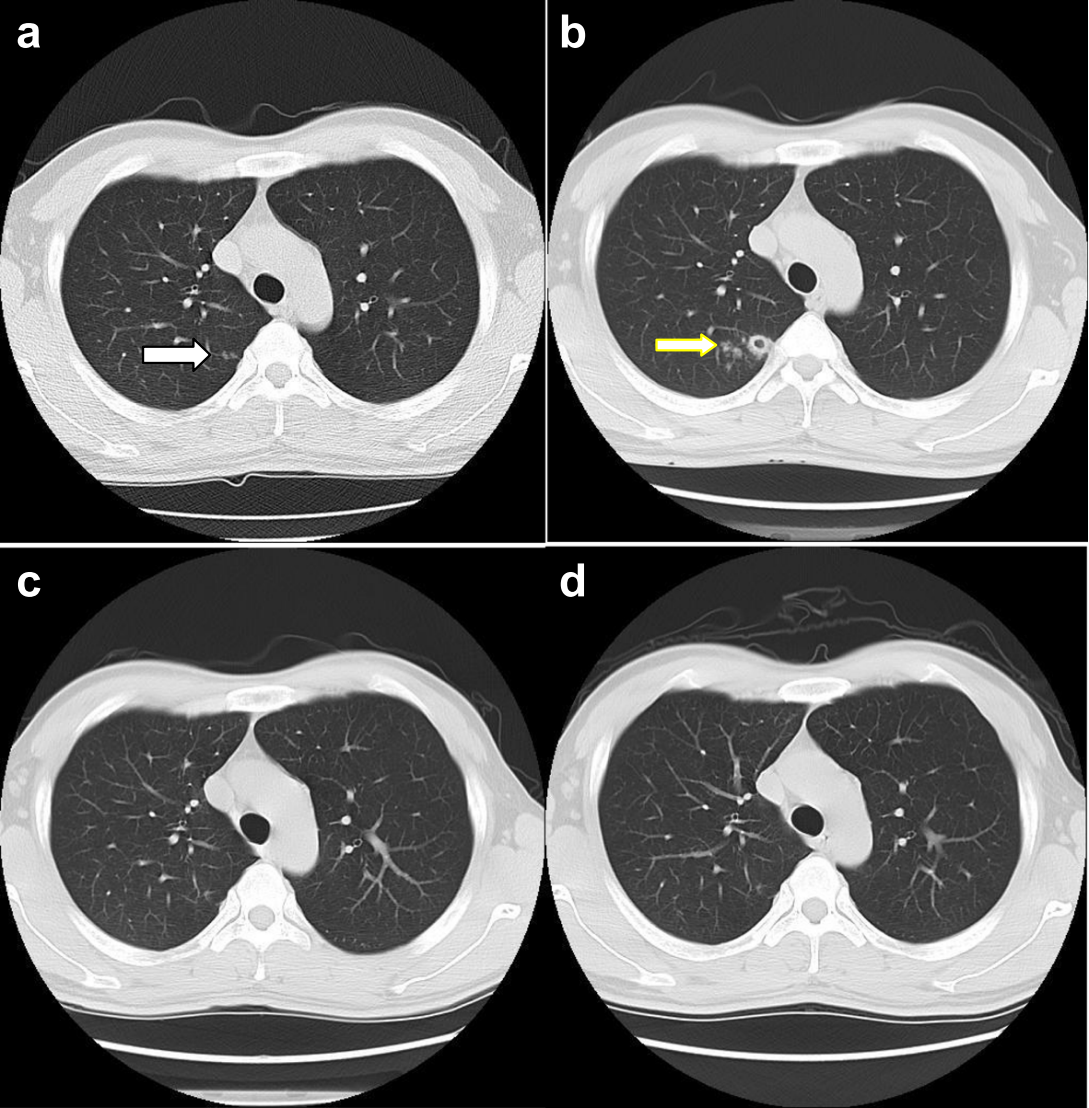
**a**-**d** LDCT images of an active PTB case (Case 3) from 2012 to 2015

A spiral CT from case 3 (Fig. 4a-d) illustrates active PTB in the right upper lung of a 36-year-old male. The chest CT scans (a – d) illustrate the CT manifestations from 2012 to 2015. Two micronodules (a, white arrow) at the pulmonary window were observed in the right upper posterior segment in 2012. We recommended anti-inflammatory treatment and ruled out active TB, but the patient did not receive any anti-inflammatory treatment. A small thickened wall cavitation with many nearby micronodules (b, yellow arrow) was observed at the same location in 2013. The final diagnosis was active TB. The cavitation was closed, and the micronodules disappeared or shrank by 2014 after one year of treatment (c). One micronodule and fibrous shadow were observed in 2015 were seen the same as in 2014 (d).

## Discussion

For the first time, this study investigated the prevalence and departmental distributions of HCWs with active PTB in a hospital specializing in treating TB patients based on LDCT examinations. The incidence and prevalence rate of active TB per year were > 2.8 times and > 4.1 times greater than the average incidence and prevalence of TB in China in 2014, respectively. In 2014, China’s TB incidence and prevalence rate were 68/100 000 and 98/100 000, respectively [2]. These observations are not surprising, given the relatively large population of TB patients seen in our hospital. There are over 100 000 TB-related outpatient services performed annually, and 2 600 TB-related patients are hospitalized. Frequent exposure to mycobacterium TB leaves HCWs at a high risk of infection with TB [6, 15–18].

According to our analysis (Fig. 1), most HCWs with active PTB (78.9%,15/19) worked in the high-risk areas. These HCWs were in frequent and close contact with TB patients and included 7 nurses and 6 physicians working in the outpatient division, TB ward and intensive care unit in addition to one CT room technician and one physician working in the oncology department who performed outpatient work with TB patients. However, only one HCW with active TB worked in an intermediate-risk area (laboratory), and three HCWs worked in low-risk areas. Tudor et al. [19] conducted a case–control study of HCWs who were diagnosed with TB in 3 district hospitals with specialized multidrug-resistant TB wards in KwaZulu-Natal, South Africa and found that cases occurred more frequently among the clinical staff (46%) and the support staff (35%). The study population distribution was not exactly the same as the population distribution in the present study. The reasons could be that the personnel statistics are not exactly the same (the hospital staff in our study did not include orderlies and cleaners). From Table 5, it is readily apparent that the incidence of active PTB in the high-risk areas was more than 13 times greater than that in the non-high-risk areas. In general, there is a strong need for the improvements to prevent and control infection among HCWs working in the high-risk areas of the hospital.

The average age of the discovered active PTB groups was 39 years old, and the peak occurred at 36 – 45 years old (42.2%). Moreover, the majority of these participants were women (15/19). The latter finding can be explained by the fact that the ratio of female staff to male staff was 2:1. Furthermore, middle-aged female HCWs have both household and work burdens in this society, which leaves them particularly vulnerable to TB infection. As noted in Table 4, the distribution of the percentages of HCWs who were affected with active TB was a relatively uniform across the different number of years of employments. These data suggest that the length of employment may not be a risk factor for TB infection, i.e., every HCW was susceptible to infection by active TB. Of the 19 active TB cases in our study, 2 (10.5%) had multidrug-resistant TB, which suggests a relatively high incidence among HCWs in TB hospitals. In 2014, the incidence of drug-resistant TB cases was estimated to be 5 – 7% in China [2]. Delays in the diagnoses, less effective treatments for drug-resistant TB, and longer periods of contact with drug-resistant TB patients may have increased the potential for the transmission of drug-resistant strains to HCWs. Accordingly, HCWs are up to six times more likely to be hospitalized for drug-resistant TB than the population they care for [20, 21].

In current practice, the diagnosis of active TB primarily relies on bacteriological inspection and X-ray detection. Among pulmonary TB cases, 58% were bacteriologically confirmed (as opposed to clinically diagnosed) in 2014. This data was unchanged from 2013 [2]. Chest radiography (CXR) has been used for over a century to diagnose pulmonary TB. However, CXR is limited by modest specificity and high interobserver variability [22]. CXR presents a low yield in the detection of active TB [23].

Computed tomography (CT) is a corroborative imaging modality for the study of TB [24]. CT helps to distinguish between active and inactive disease and is more sensitive than CXR in the detection of both localized and disseminated disease and mediastinal lymphadenopathy [12, 21, 25]. Chest CT can effectively detect 80% of patients with active PTB and 89% of those with inactive PTB [26]. Compared to conventional-dose CT, the use of LDCT for active tuberculosis detection can obviously reduce the radiation exposure and damage to the body. However, the diagnosis of TB cannot be established by radiography alone. Results from 425 individuals from 6 different European centres revealed that the sensitivities of the novel tests TST, QuantiFERON-TB GOLD, In-TubeQuanti (QFT-GIT) and T-SPOT.TB were 73.1%, 85.3%, 78.1%, and 85.2%, respectively, and the specificities were 70.6%, 48.0%, 61.9% and 44.3%, respectively [27]. In a contact investigation of a TB outbreak in a high school [28], TST and CXR were performed on all 1 044 employees and students. QFT-G was performed on the TST-positive subjects, and CT was performed on the QFT-G-positive subjects and students with TST values of ≥ 20 mm. The results revealed TST positivity in 388 subjects (37.2%), while the QFT-G tests were positive in 7.6% of the subjects (30/394). CXR exhibited abnormal findings for TB in 10 (1.0%) subjects, all of whom were TST-positive, and six of whom were QFT-G-positive. Active PTB was noted in 17 (32.7%) of 52 subjects by CT. Collectively, among 21 (1.1%) TB patients, all were TST-positive, 12 (57.1%) were QFT-G-positive, and active TB was diagnosed by CT and not by CXR in 11 subjects. Our hospital is a specialized TB hospital. The working staff has close contact with the TB patients. Therefore, it is inadequate to use TST to screen for active TB.

From the LDCT results from the patients with active PTB, tree-in-bud opacities, and micronodules, the ground glass density shadows and lymph node enlargements were found to be small lesions that were of low density and/or were hidden within the mediastinum. Such lesions are easily missed by CXR but can be delineated by LDCT. The thick wall cavity and tree-in-bud opacities are the image features of active PTB, whereas the fibrous lesions and calcification are features of inactive TB. The three cases also demonstrated the importance of annual physical examinations and the significance of dynamic changes on LDCT for the differentiation of active and inactive TB.

Lew et al. demonstrated that no diagnostic test has 100% sensitivity for TB diagnosis and suggested a combined diagnostic approach that includes TST, CXR, IGRA, and CT [29].

Kowada A et al. found that a strategy involving QFT followed by HRCT yielded the greatest benefit at the lowest cost ($US 6308.65; 27.56045 quality-adjusted life-years [QALYs]) [year 2012 values]. Cost-effectiveness was found to be sensitive to the BCG vaccination rate. HRCT chest imaging in the place of CXR is recommended as a cost-effective addition to the evaluation and management of TB contacts in public health policy [30]. In our practice, if the LDCT images revealed abnormalities in the HCWs working in high-risk areas, then active PTB would be suspected. Some of these HCWs exhibited no apparent symptoms, and some had only minor coughs in the present study. Using the final comprehensive diagnosis as the reference, the sensitivity of LDCT alone was 100%, which clearly demonstrates the importance of LDCT in the annual check-up for TB among high-risk health professionals.

There are a few limitations to this retrospective study. Not all HCWs participated in the physical examinations annually. Some of the young HCWs with relatively few years of employment and those working in low-risk areas may not have participated in the annual physical examinations. This fact may have influenced the determination of the distributions of active PTB according to age and working area. In our physical examination, LDCT scans, rather than HRCT scans, were performed to evaluate micronodules and tree-in-bud opacities, which may have resulted in an underestimation of the presence of tiny cavitations or micronodules. Nonetheless, the resolution of LDCT has steadily improved, and this improvement will facilitate the evaluation of subtle abnormalities. Moreover, although all the staff did not participate in the LDCT examinations, the annual participation rates reached 75 – 84% among all hospital staff in those years year, and these values are sufficient to represent the active tuberculosis prevalence and incidence among the hospital HCWs. Additionally, the use of even lower-dose LDCT to detect TB remains an interesting research topic, and the comparison of the performance of QFT with LDCT images is an alternative in the future.

## Conclusions

Due to their close contact with TB patients, the HCWs in a hospital specializing in the treatment of TB were at a high risk for active PTB. Annual LDCT examinations were important for discovering active PTB and distinguishing active from inactive TB disease. The government and hospital should adopt better prevention measures to protect HCWs.

## Additional file

Additional file 1: Multilingual abstracts in the five official working languages of the United Nations. (PDF 765 kb)

## Abbreviations

CXR: Chest radiography
HCWs: Healthcare workers
IGRA: Interferon-gamma release assay
LDCT: Low-dose computer tomography
PTB: Pulmonary tuberculosis
QFT: QuantiFERON-TB
TB: Tuberculosis
TST: Tuberculin skin test
WHO: World Health Organization
