# Supplementary material for: Use of low-dose computed tomography to assess pulmonary tuberculosis among healthcare workers in a tuberculosis hospital
Source: Infect Dis Poverty. 2017 Mar 24;6:68. doi: 10.1186/s40249-017-0274-6 (PMC5364637; doi:10.1186/s40249-017-0274-6)
Supplement: Additional file 1: — Multilingual abstracts in the five official working languages of the United Nations. (PDF 765 kb) [file 40249_2017_274_MOESM1_ESM.pdf]

Translation of the abstract into the five official working languages of the United Nations

استخدام صور الأشعة المقطعية منخفضة الإشعاع لتقييم مرض السل الرئوي بين العاملين في الرعاية الصحية في مستشفى للسل

وى هي، بو دونج تشن يان لف، تشن تشو جين بينج شو بينج شين لف، شين هوا تشو، فنج قانج نينج، تشنج لى هاى دونج-بو وانج، جي تشنج،

ملخص
خلفية: وفقا لمنظمة الصحة العالمية، الصين واحدة من 22 دولة توجد بها عدوى السل الخطير وواحدة من 27 دولة توجد بها سلالات السل الخطيرة المقاومة للأدوية المتعددة. وعلى الرغم من تراجع مرض السل في العدد الكلي للسكان، لا يزال العاملون في الرعاية الصحية عرضة بدرجة عالية لمخاطر الإصابة. مقارنة مع البلدان ذات الدخل المرتفع، ينتشر السل بين العاملين في الرعاية الصحية بنسبة أعلى في البلدان المنخفضة والمتوسطة الدخل. أصبحت صور الأشعة المقطعية منخفضة الإشعاع (LDCT) أكثر شعبية نظرا لحساسيتها العالية وانخفاض جرعة إشعاع فيها. ومع ذلك، لم تكن هناك أي تقارير عن مرض السل الرئوي النشط (PTB) بين العاملين في الرعاية الصحية التي تم تقييمها باستخدام صور الأشعة المقطعية منخفضة الإشعاع. هدفت هذه الدراسة إلى دراسة أوضاع مرض السل الرئوي في العاملين في الرعاية الصحية في المستشفيات المتخصصة في علاج السل واستكشاف أهمية تطبيق صور الأشعة المقطعية منخفضة الإشعاع على هؤلاء العاملين.
الأساليب: قامت هذه دراسة بتحليل بأثر رجعي بيانات الفحص البدني للعاملين في الرعاية الصحية في مستشفى بكين للأمراض الصدرية من سبتمبر 2012 إلى ديسمبر 2015. تم إجراء فحص صور الأشعة المقطعية منخفضة الإشعاع لجميع الحالات. تم إجراء مقارنات بين مرض السل الرئوي في العاملين وغير العاملين وفقا لنتائج التصوير المقطعي باستخدام اختبار بيرسون خي مربع أو اختبار الدقة لفيشر. أجريت مقارنات بين حالات مرض السل الرئوي النشط في المناطق المعرضة للخطر والمناطق غير المعرضة للخطر باستخدام اختبار بيرسون خي مربع. كانت التحاليل التي أجريت على مرض السل الرئوي النشط وفقا لمختلف الأعمار، وعدد سنوات في الوظيفة، ومخاطر مجالات العمل. وتمت مقارنة مرض السل الرئوي النشط وفقا لتشخيصه عن طريق اختبارات صور الأشعة المقطعية منخفضة الإشعاع منفردا مع التشخيص الشامل والنهائي، وحسبت الحساسية والقيمة التنبؤية الإيجابية.
النتائج: تم إدراج ما مجموعه 1012 من المشاركين في هذه الدراسة. خلال فترة 4 سنوات من الفحوصات الطبية، وجد مرض السل الرئوي النشط في 19 حالة، ووجد مرض السل الرئوي غير النشط في 109 حالات. وكان انتشار مرض السل الرئوي النشط في المشاركين 1.24٪، 0.67٪، 0.81٪، 0.53٪ للسنوات 2012 إلى عام 2015. وكانت الحالات المماثلة من مرض السل الرئوي النشط بين المشاركين في مستشفى السل بنسبة 0.86٪، 0.41٪، 0.54٪، و0.26 ٪. معظم العاملين في الرعاية الصحية المصابين بالسل النشط (78.9٪، 19/15) عملوا في المناطق المعرضة للخطر من المستشفى. كان هناك اختلاف كبير في حالات مرض السل الرئوي النشط بين العاملين في الرعاية الصحية الذين عملوا في المناطق ذات المخاطر العالية والمناطق غير المعرضة للخطر (نسبة الأرجحية [OR]، 14.415؛ فاصل الثقة 95٪، 733، 4-896، 43). وكشفت المقارنات بين العلامات في الصور المقطعية بين المجموعات النشطة وغير النشطة عن طريق اختبارات خي مربع أن علامة شجرة في برعم، والظل ليفي، والتكلس أظهرت فروق ذات دلالة إحصائية (P = 0.000، 0.021، 0.001، و0.024 على التوالي). علامة شجرة في برعم وعتامة التجويف تظهر السل الرئوي النشط، في حين أن الظل الليفي وعتامة التكلس هي الملامح الرئيسية لمرض السل الرئوي غير نشط. وكشفت مقارنة مع التشخيصات الشاملة النهائية أن الحساسية والقيمة التنبؤية الإيجابية للتشخيص في مرض السل الرئوي النشط على أساس صور الأشعة المقطعية منخفضة الإشعاع وحدها كانت 100٪ و86.4٪ على التوالي.
الخلاصة: عمال الرعاية الصحية في المستشفيات السل هم مجموعة معرضة بنسبة عالية لمخاطر الإصابة بمرض السل الرئوي النشط. إجراء اختبارات صور الأشعة المقطعية منخفضة الإشعاع بصورة سنوية لهذه المجموعات المعرضة للخطر ممكن وضروري.

Translated from English version into Arabic by Mahmoud Sami, through

TRANSLATORS
WITHOUT BORDERS

低剂量 CT 扫描对一个结核病医院医务工作者肺结核情况的评估

Wei He, Bu-Dong Chen, Yan Lv, Zhen Zhou, Jin-Ping Xu, Ping-Xin Lv, Xin-Hua Zhou, Feng-Gang Ning, Cheng-Hai Li, Dong-Po Wang and Jie Zheng

引言：根据世界卫生组织报道，中国既是全球 22 个肺结核高发病率国家之一，也是全球 27 个耐药肺结核发生率高的国家之一。尽管全球结核病的总人数下降，但是医务工作者(HCWs)仍是结核感染的高风险人群。与高收入国家相比，医务工作者的结核病患病率在低收入和中

等收入国家更高。低剂量 CT 具有较高的灵敏度和较低的辐射剂量，越来越受到欢迎。然而，有关低剂量 CT 检查对医务工作者检查活动性肺结核的研究目前尚未见报道。本研究的目的是研究结核病专科医院中医务人员肺结核的状况，并探讨低剂量 CT 扫描在这些人群中的意义。
方法：本研究回顾性分析了北京胸科医院从 2012 年 9 月到 2015 年 12 月医务工作者的健康体检数据。所有参与者均行肺部低剂量 CT 检查。采用 Pearson 卡方检验或确切概率法比较活动性和非活动性肺结核的 CT 表现。采用 Pearson 卡方检验比较高风险区域和非高风险区域之间活动性肺结核的发病率。根据年龄,工作岗位和工作领域风险的不同对活动性肺结核的发病进行分析。比较仅以 LDCT 检查诊断活动性肺结核和最终综合诊断活动性肺结核的差别，并计算敏感性和阳性预测值。
结果：共有 1012 名参与者被纳入本研究。在四年体检中,共发现 19 例活动性肺结核和 109 例非活动性肺结核。从 2102 年到 2015 年，参与人员中活性肺结核的患病率分别为每年 1.24%，0.67%，0.81%和 0.53%；发病率分别为 0.86%,0.41%,0.54%,0.26%。大多数活动性肺结核病人(15/19，78.9%)在医院的高风险区域工作。活动性肺结核发病率，在高风险区域和非高风险区域工作有显著差异 (优势比为 14.415，95%置信区间为 4.733 —43.896)。通过卡方检验比较活动性肺结核组和非活动性肺结核组的 CT 表现，提示树芽征，空洞，纤维性病变和钙化有显著性差异(P 值分别为 0.000,0.021,0.001 及 0.024)。与最终的综合诊断相比，仅靠低剂量 CT 扫描诊断活动性肺结核的灵敏度和阳性预测值活性肺结核仅分别为 100%和 86.4%。
结论：结核病医院的医务工作者是活动性肺结核的高危人群。在这些高危人群，每年行低剂量 CT 检查是可行和必要的。

Translated from English version into Chinese by Wei He

Utilisation de la tomodensitométrie à faible dose pour l'évaluation de la tuberculose pulmonaire parmi le personnel médical d'un hôpital spécialisé dans le traitement de la tuberculose

Wei He, Bu-Dong Chen, Yan Lv, Zhen Zhou, Jin-Ping Xu, Ping-Xin Lv, Xin-Hua Zhou, Feng-Gang Ning, Cheng-Hai Li, Dong-Po Wang and Jie Zheng,

Résumé
Contexte : Selon l'Organisation mondiale de la Santé, la Chine est l'un des 22 pays affecté par des infections tuberculeuses graves et l'un des 27 pays abritant des souches multirésistantes préoccupantes. Malgré le recul de la tuberculose dans la population générale, le personnel médical reste exposé à un fort risque d'infection. La prévalence de la tuberculose dans ces professions est plus importante dans les pays à bas et moyens revenus que dans ceux à revenus élevés. La tomodensitométrie à faible dose (TDM-FD) est en expansion en raison de sa très bonne sensibilité et de sa dose d'irradiation relativement faible. Il n'existe cependant pas de rapports de son utilisation pour l'évaluation de la tuberculose pulmonaire (TP) active parmi les professions de santé. Le but de cette étude était d'examiner le statut tuberculeux des personnels médicaux d'hôpitaux spécialisés dans le traitement de la tuberculose et d'explorer l'intérêt de la TDM-FD pour ces personnels.
Méthodes : Cette étude a analysé rétrospectivement les données d'examens de santé physique de membres du personnel de l'Hôpital de pneumologie de Beijing, relevées de septembre 2012 à décembre 2015. Des examens des poumons par TDM à faible dose ont été réalisés dans tous les cas. Les comparaisons entre les TP actives et inactives selon les résultats de TDM ont été faites à l'aide du test de chi-deux de Pearson ou du test exact de Fisher. Les comparaisons entre l'incidence de la TP active dans les zones à haut risque et non à risque ont été réalisées avec le test de chi-deux de Pearson. Des analyses de la TP active ont été effectuées en fonction de l'âge, de l'ancienneté au poste et des risques des domaines de travail. La TP active diagnostiquée uniquement par l'examen de TDM-FD a été comparée avec les diagnostics d'ensemble finaux et la sensibilité ainsi que la valeur prédictive positive de la méthode ont été calculées.
Résultats : Au total, 1012 participants ont été inclus dans l'étude. Sur ces 4 ans d'examens médicaux, une TP active a été décelée dans 19 cas et une TP inactive dans 109. La prévalence de la

TP active parmi les participants était de 1,24 %, 0,67 %, 0,81 % et 0,53 % pour les années 2012 à 2015. Les incidences correspondantes parmi le personnel de l'hôpital pneumologique étaient de 0,86 %, 0,41 %, 0,54 % et 0,26 %. La plupart des professionnels de la santé atteints d'une TP active (78,9 %, 15 sur 19) travaillaient dans des secteurs à haut risque de l'hôpital. On constate une différence significative dans l'incidence de la TP active entre le personnel travaillant dans les secteurs à risque et d'autres secteurs (rapport de cotes [*OR*] 14,415, intervalle de confiance à 95 % de 4733 à 43 896). La comparaison des signes à la TDM entre les groupes de TP active et inactive par le test de chi-deux a mis en évidence des différences significatives pour les images d'arbre bourgeonnant, de cavités, d'ombres fibreuses et de calcification (P = 0,000, 0,021, 0,001 et 0,024, respectivement). Les opacités en arbre bourgeonnant et cavitaires suggèrent une tuberculose pulmonaire active, tandis que les ombres fibreuses et les calcifications sont les caractéristiques principales de la TP inactive. La comparaison avec le diagnostic d'ensemble final a montré que la sensibilité et la valeur prédictive positive du diagnostic de TP active basé sur la TDM-FD seule étaient respectivement de 100 % et 86,4 %.
Conclusion : Le personnel médical des hôpitaux assurant le traitement de la tuberculose est exposé à un risque important de tuberculose pulmonaire active. Des examens annuels par TDM-FD de ce groupe à risques sont possibles et nécessaires.

Translated from English version into French by Suzanne Assenat, through

Использование компьютерной томографии с низкой дозой облучения для оценки распространенности туберкулеза легких среди медицинских работников туберкулезной больницы

Вэй Хэ (Wei He), Будун Чэнь (Bu-Dong Chen), Янь Лв (Yan Lv), Чжэнь Чжоу (Zhen Zhou), Цзиньпин Сюй (Jin-Ping Xu), Пинсинь Лв (Ping-Xin Lv), Синьхуа Чжоу (Xin-Hua Zhou), Фэнган Нин (Feng-Gang Ning), Чэнхай Ли (Cheng-Hai Li), Дунпо Ван (Dong-Po Wang), Цзе Чжэн (Jie Zheng)

Реферат

Предыстория: По данным Всемирной организации здравоохранения, Китай является одной из 22 стран с серьезной заболеваемостью туберкулезом (ТБ) и одной из 27 стран со значительным количеством штаммов туберкулеза, резистентных ко многим лекарственным препаратам. Несмотря на снижение заболеваемости туберкулезом среди населения в целом, медицинские работники по-прежнему подвергаются большому риску заражения туберкулезом. По сравнению со странами с высоким уровнем дохода, отмечается более высокая распространенность ТБ среди медицинских работников в странах с низким и средним доходом. Компьютерная томография с низкой дозой облучения (КТНД) становится все более популярной благодаря ее исключительной чувствительности и более низким дозам облучения. Однако, нет никаких сведений об оценке распространенности активной формы туберкулеза легких среди медицинских работников, проведенной с помощью компьютерной томографии с низкой дозой облучения. Цели данной научной работы – исследование состояния туберкулеза легких среди медицинских работников в больницах, специализирующихся на лечении туберкулеза, и изучение значимости использования компьютерной томографии с низкой дозой облучения для обследования этих работников.
Методы: В исследовании ретроспективно проанализированы данные медицинского осмотра работников пекинской больницы грудной хирургии за период с сентября 2010 года по декабрь 2015 года. Во всех случаях была проведено КТ-обследование легких. На основе данных компьютерной томографии проводилось сравнение активной и неактивной форм туберкулеза легких с применением критерия согласия Пирсона или точного теста Фишера. Сравнение заболеваемости активной формы ТБ легких на участках повышенной и невысокой степени

риска выполнялось по критерию согласия Пирсона. Анализ активной формы туберкулеза легких проводился с учетом возраста, количества лет на данном рабочем месте и рисков на рабочих участках. Проведено сравнение диагноза активной формы ТБ легких, поставленного только методом КТНД, с окончательным комплексным диагнозом; рассчитана прогностическая ценность чувствительности и положительного результата.
Результаты: В исследовании участвовало всего 1012 человек. За четырехлетний период медицинских обследований выявлено 19 случаев активного и 109 неактивного туберкулеза легких. Распространенность активного ТБ легких среди участников составила 1,24%; 0,67%; 0,81% и 0,53% за период с 2012 по 2015 годы. Соответствующая заболеваемость активной формой ТБ легких среди участников туберкулезной больницы составила 0,86%; 0,41%; 0,54% и 0,26%. Большинство медицинских работников с активным ТБ легких (78,9%, 15/19) были заняты в больнице на участках повышенного риска. Отмечено существенное различие в распространенности активного туберкулеза легких среди медицинских работников, работавших на участках повышенного и невысокого риска (отношение вероятностей 14,415; 95%-й доверительный интервал 4,733-43,896). Сопоставление КТ-симптомов у активной и неактивной групп с применением критерия согласия обнаружило существенную разницу (*P* = 0,000; 0,021; 0,001 и 0,024 соответственно) в таких симптомах как «дерево в почках», каверна, фиброзная тень и кальцификация. Тени «дерева в почках» и каверны предполагают активную форму туберкулеза легких, тогда как фиброзная тень и кальцинированные затемнения являются основными свойствами неактивного туберкулеза легких. Сравнение с окончательным комплексным диагнозом показало, что прогностическая ценность чувствительности и положительного результата диагноза активного ТБ легких на основе КТ с низкой дозой облучения составила 100% и 86,4% соответственно.
Вывод: Медицинские работники туберкулезных больниц находятся в группе повышенного риска заболеваемости активной формой ТБ легких. Целесообразно и необходимо ежегодное обследование таких групп методом КТ с низкой дозой облучения.

Translated from English version into Russian by Tatyana Johnson, through

TRANSLATORS
WITHOUT BORDERS

Utilización de la tomografía computarizada de baja dosis para evaluar la tuberculosis pulmonar en los profesionales de la salud que trabajan en un hospital para tuberculosos

Wei He, Bu-Dong Chen, Yan Lv, Zhen Zhou, Jin-Ping Xu, Ping-Xin Lv, Xin-Hua Zhou, Feng-Gang Ning, Cheng-Hai Li, Dong-Po Wang and Jie Zheng.

Resumen
Antecedentes: según la Organización Mundial de la Salud, China es uno de los 22 países con infecciones graves por tuberculosis (TB) y uno de los 27 países con graves cepas de TB resistentes a múltiples medicamentos. Aunque los casos de tuberculosis han disminuido en toda la población, los profesionales de la salud (HCW, por sus siglas en inglés) continúan estando expuestos a un alto riesgo de infección. En comparación, la prevalencia de TB entre los HCW es mayor en los países de ingresos bajos y medios que en los de altos ingresos. La tomografía computarizada de baja dosis (LDCT, por sus siglas en inglés) está ganando popularidad debido a su sensibilidad superior y a su menor dosis de radiación. No obstante, no se han registrado casos de tuberculosis pulmonar (PTB, por sus siglas en inglés) activa entre los HCW, según lo evaluado por la LDCT. El objetivo de este estudio era examinar la situación de la PTB en los HCW que trabajan en hospitales que se especializan en el tratamiento de la tuberculosis y explorar la relevancia de aplicar la LDCT a estos trabajadores.
Métodos: en este estudio se analizaron, en forma retrospectiva, los datos de los exámenes físicos de los profesionales de la salud del Beijing Chest Hospital, desde setiembre de 2012 a diciembre de 2015. Se realizaron exámenes de pulmón con TC de baja dosis en todos los casos. En función de los

hallazgos de la TC, se hicieron comparaciones entre la PTB activa e inactiva por medio de la prueba ji cuadrado o prueba exacta de Fisher. Las comparaciones entre las incidencias de PTB activa en áreas con o sin alto riesgo se llevaron a cabo por medio de la prueba ji cuadrado de Pearson. Los análisis de PTB activa se realizaron en función de las distintas edades, la antigüedad en el trabajo y los riesgos en las áreas de trabajo. Se comparó la PTB activa, conforme a lo diagnosticado por los exámenes de la LDCT solamente, con los diagnósticos completos y definitivos; y se calcularon el valor predictivo positivo y la sensibilidad.

Resultados: en este estudio se incluyó a un total de 1.012 participantes. En el periodo de 4 años de exámenes médicos, se encontró PTB activa en 19 casos y PTB inactiva en 109 casos. Desde 2012 a 2015, la prevalencia de PTB activa en los participantes fue de 1,24 %; 0,67 %; 0,81 % y 0,53 %. Las incidencias correspondientes de PTB activa entre los participantes del hospital para tuberculosos fueron 0,86 %; 0,41 %; 0,54 % y 0,26 %. La mayoría de los HCW con TB activa (78,9 %, 15/19) trabajaban en áreas con alto riesgo del hospital. Hubo una diferencia significativa en las incidencias de PTB activa entre los HCW que trabajaban en las áreas con y sin alto riesgo (odds ratio [OR], 14,415; intervalo de confianza al 95 %, 4,733-43,896). Las comparaciones de los signos de la TC entre grupos activos e inactivos, realizadas por medio de las pruebas ji cuadrado, revelaron que los signos de árbol en brote, cavidad, sombra fibrosa y calcificación mostraban diferencias significativas (P = 0,000; 0,021; 0,001 y 0,024; respectivamente). Las opacidades de tipo árbol en brote y cavidad sugieren tuberculosis pulmonar activa, mientras que las opacidades de tipo sombra fibrosa y calcificación son características principales de la tuberculosis pulmonar inactiva. La comparación efectuada con los diagnósticos completos y definitivos reveló que el valor predictivo positivo y la sensibilidad de los diagnósticos de PTB activa, basados solamente en la LDCT, fueron 100 % y 86,4 %, respectivamente.

Conclusión: los profesionales de la salud que trabajan en hospitales para tuberculosos conforman un grupo con alto riesgo de padecer PTB activa. Es factible y necesario realizar anualmente exámenes por LDCT a dichos grupos con alto riesgo.

Translated from English version into Spanish by dsp3112, through

TRANSLATORS
WITHOUT BORDERS
